# Clinical Management and Outcomes of Ectopic Pregnancies: Experience From Sohar Hospital Over Three Years

**DOI:** 10.7759/cureus.95451

**Published:** 2025-10-26

**Authors:** Samiha Samad, Kaukab Tashfeen, Naseema Anjum, Sabira Khuda Bakhsh, Sheeba Burney, Sakina Noushad Saleem

**Affiliations:** 1 Obstetrics and Gynaecology, Sohar Hospital, Sohar, OMN; 2 Obstetrics and Gynaecology, Bugshan Hospital, Jeddah, SAU; 3 Medicine, Dow University of Health Sciences, Karachi, PAK; 4 Medicine, Tbilisi State Medical University, Tbilisi, GEO

**Keywords:** ectopic pregnancy, fertility, medical management, surgical management, treatment outcome

## Abstract

Background

Ectopic pregnancy is a potentially life-threatening condition requiring timely diagnosis and appropriate management. This study aims to evaluate the clinical presentation, management strategies, and treatment outcomes of women with ectopic pregnancy at a tertiary care hospital in Oman over a three-year period.

Materials and methods

We retrospectively reviewed 404 ectopic pregnancy cases from 2020 to 2022. Patients received methotrexate or surgical treatment. Patients were evaluated for success rate, complications, hospital stay, and need for further intervention using t-tests, chi-square, and multivariate models.

Results

No significant differences were found between groups regarding age, gravidity, parity, prior ectopic pregnancy, pelvic inflammatory disease, or pelvic surgery. Clinical presentations were similar, though hemodynamic instability was more frequent in the surgical group (15.3% vs. 6.4%, p = 0.01), independently predicting surgical intervention (adjusted odds ratio (AOR) = 2.65, 95% CI: 1.22-5.75). Surgical management yielded a higher success rate (97.6% vs. 85.9%, p < 0.001; AOR = 4.85, 95% CI: 2.01-11.7). Laparoscopy was the predominant surgical method (79.8%), while 28.8% of medically treated patients received multidose methotrexate. Complication rates were comparable (7.7% vs. 7.3%, p = 0.88), but hospital stay was longer in the surgical group (4.8 vs. 2.4 days, p < 0.001). Additional interventions were more common in the medical group (5.1% vs. 0.8%, p = 0.016; AOR = 6.78, 95% CI: 1.35-33.9). Tubal ectopic pregnancies predominated (81.2%), with treatment modality significantly associated with ectopic site (p = 0.0239). Management type was significantly linked to treatment outcome (χ² = 14.5, p < 0.001).

Conclusion

Surgical management demonstrates higher efficacy and reduced need for retreatment, particularly in hemodynamically unstable or non-tubal ectopic pregnancies. Treatment decisions should be guided by clinical stability and anatomical site to optimize outcomes.

## Introduction

Ectopic pregnancy, defined as the implantation of a fertilized ovum outside the uterine cavity, remains a significant cause of maternal morbidity and mortality worldwide. Despite advances in diagnostic imaging and medical therapy, ectopic pregnancies account for approximately 1-2% of all pregnancies and up to 10% of pregnancy-related deaths in the first trimester [1-3]. The clinical presentation can vary widely, ranging from mild abdominal discomfort to life-threatening hemorrhage, making early diagnosis and timely management critical. Historically, the management of ectopic pregnancy relied heavily on surgical intervention [4].

The development of medical treatment with methotrexate (MTX) has expanded therapeutic options, particularly for hemodynamically stable patients with early, unruptured ectopic pregnancies [5]. Medical management offers the advantages of preserving tubal function, reducing hospital stay, and minimizing surgical risks. Nevertheless, careful patient selection, serial beta-human chorionic gonadotropin (β-hCG) monitoring, and close follow-up are essential to ensure treatment success and prevent complications [6,7]. The choice between medical and surgical management depends on several factors, including the patient’s clinical stability, ectopic mass size and location, β-hCG levels, previous reproductive history, and the presence of comorbidities. Studies have demonstrated that while surgical management provides higher immediate success rates, medical management remains an effective and safe alternative in selected cases [1,4,8].

Multivariate analyses have identified several key risk factors associated with treatment failure in ectopic pregnancy, particularly among patients managed with MTX. Elevated pre-treatment β-hCG levels have consistently been linked to a higher risk of MTX failure, with studies reporting that higher initial β-hCG values significantly predict unsuccessful medical management [9]. Similarly, the size of the ectopic mass is an important predictor, as larger masses (>3.5 cm) are associated with increased failure rates, while smaller masses respond more successfully to single-dose MTX therapy [10]. A history of pelvic inflammatory disease (PID) also increases the risk of treatment failure, with odds ratios (ORs) suggesting a three- to four-fold higher likelihood of unsuccessful medical management [11]. These findings highlight the importance of early identification of high-risk patients based on clinical and laboratory parameters, enabling individualized management strategies that can optimize outcomes and reduce the need for surgical intervention.

Globally, evaluating real-world data on ectopic pregnancy management helps optimize patient care, guide resource allocation, and improve clinical outcomes through insights from tertiary care analyses. In Oman, limited data exist regarding the epidemiology and clinical outcomes of ectopic pregnancy. Sohar Hospital, a high-volume tertiary care center in northern Oman, serves a large population and manages a significant proportion of reproductive-aged women presenting with gynecological emergencies. Studying the clinical experience of ectopic pregnancy management in this setting provides critical local evidence, helps identify population-specific risk factors, and informs best practices tailored to the Omani healthcare system [5,12]. Such context-specific data are essential for developing national guidelines, optimizing resource use, and improving maternal health outcomes across the country. The objective of this study was to evaluate the clinical presentation, management strategies, and outcomes of women with ectopic pregnancy at Sohar Hospital over a three-year period, providing evidence to guide optimal care in the Omani healthcare context.

## Materials and methods

Study design and setting

This retrospective observational study was conducted at Sohar Hospital, Oman, over a three-year period from January 2020 to December 2022. The study aimed to evaluate the clinical presentation, management strategies, and outcomes of women diagnosed with ectopic pregnancy in a tertiary care setting. All patient identifiers were anonymized, and data confidentiality was maintained in accordance with institutional and international ethical research standards.

Study population

A total of 412 women diagnosed with ectopic pregnancy during the study period were initially identified from hospital records. After applying the inclusion and exclusion criteria, 404 patients met the eligibility criteria and were included in the final analysis.

Inclusion criteria

Women of reproductive age (15-49 years) with a confirmed diagnosis of ectopic pregnancy based on clinical evaluation, transvaginal ultrasonography, and serial serum β-hCG measurements were included.

Exclusion criteria

Patients were excluded if they had incomplete medical records, received initial management at another institution, or were lost to follow-up. These criteria were applied to minimize selection bias and ensure the accuracy of the data.

Ethical approval

Ethical clearance for this study was obtained from the Research and Ethical Committee, North Batinah Governorate (Reference No. MH/DHGS/NBG/1023208775/2019, dated 19 October 2019). All procedures followed the ethical principles outlined in the Declaration of Helsinki (2013 revision).

Data collection

Data were collected retrospectively from patient medical records using a pre-designed and pre-tested structured data collection form to ensure uniformity and minimize extraction errors (Appendices). Two trained researchers independently extracted data, and discrepancies were resolved through discussion and consensus. The collected data included demographic and obstetric characteristics (age, gravidity, parity, history of previous ectopic pregnancy, PID, prior pelvic surgery, and use of assisted reproductive techniques). Clinical presentation was assessed by documenting abdominal pain characteristics, vaginal bleeding, syncope, hemodynamic status, and vital signs upon admission.

Laboratory findings included hemoglobin levels, white blood cell counts, and serial serum β-hCG measurements. Imaging findings from transvaginal ultrasonography were reviewed for the site and size of the ectopic mass, presence of free peritoneal fluid, and signs of rupture.

Hemodynamic instability was defined as systolic blood pressure <90 mmHg or pulse rate >110 bpm, while ectopic rupture was defined as intraoperative confirmation of tubal rupture or hemoperitoneum >500 mL. All data were entered into a secure electronic database for further statistical analysis.

Management protocol

Patients were managed according to institutional guidelines and classified into two groups based on the treatment modality: medical or surgical management. MTX was administered as a single dose (50 mg/m² IM) or multiple doses (1 mg/kg alternating with leucovorin), based on β-hCG levels and clinical stability. Surgical decisions (salpingectomy vs. salpingostomy and laparoscopy vs. laparotomy) were guided by tubal integrity, rupture status, and hemodynamic condition.

Medical management involved the administration of MTX using either single-dose or multidose protocols, depending on the patient’s initial β-hCG level, clinical stability, and sonographic findings. Details such as dosage, treatment response, and need for additional doses were recorded. Surgical management was performed in patients who were hemodynamically unstable, had contraindications to MTX, or failed medical therapy. Procedures included laparoscopic or open salpingectomy/salpingostomy, with detailed documentation of intraoperative findings, type of surgery performed, estimated hemoperitoneum volume, and perioperative complications.

Outcome measures

Post-treatment outcomes assessed included duration of hospital stay, need for further intervention, treatment-related complications, and subsequent fertility outcomes where available.

Success of medical management was defined as complete resolution without the need for surgical intervention, while surgical success was defined as the absence of intraoperative or postoperative complications and no requirement for repeat surgery.

Statistical analysis

All collected data were entered into a secure electronic database and analyzed using the Statistical Package for the Social Sciences (SPSS), version 26.0 (IBM Corp., Armonk, NY). Data were checked for completeness, accuracy, and consistency prior to analysis. Continuous variables were tested for normality using the Shapiro-Wilk test and visual histogram inspection. Normally distributed variables were expressed as mean ± standard deviation (SD), while non-normally distributed data were expressed as median (interquartile range (IQR)). Categorical variables were presented as frequencies and percentages. Comparisons between the medical and surgical management groups were performed using the Student’s t-test for continuous variables with normal distribution and the Mann-Whitney U test for non-normally distributed data. The chi-square test (χ²) or Fisher’s exact test was used for categorical variables, depending on expected frequencies. To identify independent predictors of successful treatment, binary logistic regression analysis was conducted, adjusting for potential confounders such as age, serum β-hCG level, ectopic mass size, and presence of free fluid. Variables with a p-value < 0.20 in univariate analysis were included in the multivariable model. Results were expressed as ORs with 95% confidence intervals (CIs). The level of statistical significance was set at p < 0.05 for all analyses. Missing data were managed using listwise deletion, and sensitivity analyses were conducted to confirm the robustness of the findings. Data visualization techniques, including bar charts and frequency plots, were used to present trends in presentation, management, and outcomes.

## Results

In this retrospective analysis of 404 patients diagnosed with ectopic pregnancy, there were no statistically significant differences between the medically and surgically managed groups with respect to mean age, gravidity, or parity. Similarly, previous ectopic pregnancy, history of PID, and prior pelvic surgery did not demonstrate any significant association with the management approach. The clinical presentations, including abdominal pain and vaginal bleeding, were comparable across both groups. Notably, hemodynamic instability was significantly more prevalent among surgically managed patients (15.3%) compared to those treated medically (6.4%) (p = 0.01). Multivariate analysis revealed that hemodynamic instability independently increased the likelihood of surgical intervention, with an adjusted OR of 2.65 (95% CI: 1.22-5.75) (Table 1).

**Table 1:** Comparison of clinical and demographic characteristics between medical and surgical management groups ^‡^Independent t-test applied. ^§^Chi-square test applied. ^*^p < 0.05 is considered statistically significant. AOR: adjusted odds ratio; N/A: not applicable; PID: pelvic inflammatory disease

Variables	Total (N = 404)	Medical (n = 156)	Surgical (n = 248)	Test value	p-value	AOR	95% CI
Age (years) mean ± SD	29.4 ± 5.8	28.9 ± 5.6	29.7 ± 5.9	1.45^‡^	0.15	N/A	N/A
Gravidity mean ± SD	2.1 ± 1.2	2.0 ± 1.1	2.2 ± 1.3	1.55^‡^	0.12	N/A	N/A
Parity mean ± SD	1.1 ± 1.1	1.0 ± 1.0	1.1 ± 1.2	1.21^‡^	0.22	N/A	N/A
Previous ectopic pregnancy	38 (9.4%)	12 (7.7%)	26 (10.5%)	0.78^§^	0.38	1.40	0.68-2.87
History of PID	52 (12.9%)	16 (10.3%)	36 (14.5%)	1.15^§^	0.28	1.44	0.77-2.70
Previous pelvic surgery	45 (11.1%)	14 (9.0%)	31 (12.5%)	0.92^§^	0.34	1.44	0.71-2.89
Abdominal pain	352 (87.1%)	134 (85.9%)	218 (87.9%)	0.27^§^	0.60	1.18	0.64-2.18
Vaginal bleeding	210 (52.0%)	80 (51.3%)	130 (52.4%)	0.04^§^	0.84	1.04	0.69-1.58
Hemodynamically unstable	48 (11.9%)	10 (6.4%)	38 (15.3%)	6.52^§^	0.01^*^	2.65	1.22-5.75

The overall treatment success rate was significantly higher in the surgically managed group (97.6%) compared to the medically treated group (85.9%) (p < 0.001). Patients managed surgically were nearly five times more likely to achieve treatment success (adjusted odds ratio (AOR) = 4.85, 95% CI: 2.01-11.7) than those receiving medical therapy. Among medically managed patients, 28.8% received a multidose MTX regimen, while laparoscopic surgery was the predominant approach among surgically treated patients (79.8%), followed by open surgery (20.2%). The incidence of post-treatment complications was comparable between the two groups (7.7% vs. 7.3%, p = 0.88), indicating no significant difference in safety profiles. However, the mean duration of hospital stay was significantly longer among patients undergoing surgical management (4.8 ± 2.1 days) compared to those managed medically (2.4 ± 1.2 days, p < 0.001). Furthermore, the need for additional intervention was notably higher in the medical group (5.1%) compared to the surgical group (0.8%) (p = 0.016), with an adjusted OR of 6.78 (95% CI: 1.35-33.9) (Table 2).

**Table 2:** Comparison of treatment outcomes between medical and surgical management groups ^‡^Independent t-test applied. ^§^Chi-square test applied. ^*^p < 0.05 is considered statistically significant. AOR: adjusted odds ratio; N/A: not applicable

Variables	Total (N = 404)	Medical (n = 156)	Surgical (n = 248)	Test value	p-value	AOR	95% CI
Treatment success, n (%)	376 (93.1%)	134 (85.9%)	242 (97.6%)	14.5^§^	<0.001^*^	4.85	2.01-11.7
Methotrexate multi-dose, n (%)	45 (11.1%)	45 (28.8%)	N/A	N/A	N/A	N/A	N/A
Laparoscopic surgery, n (%)	198 (49.0%)	N/A	198 (79.8%)	N/A	N/A	N/A	N/A
Open surgery, n (%)	50 (12.4%)	N/A	50 (20.2%)	N/A	N/A	N/A	N/A
Post-treatment complications, n(%)	30 (7.4%)	12 (7.7%)	18 (7.3%)	0.02^§^	0.88	0.95	0.43-2.08
Mean hospital stay - days mean ± SD	3.9 ± 2.0	2.4 ± 1.2	4.8 ± 2.1	12.6^‡^	<0.001^*^	N/A	N/A
Need for additional intervention, n (%)	10 (2.5%)	8 (5.1%)	2 (0.8%)	5.82^§^	0.016^*^	6.78	1.35-33.9

**Figure 1:**
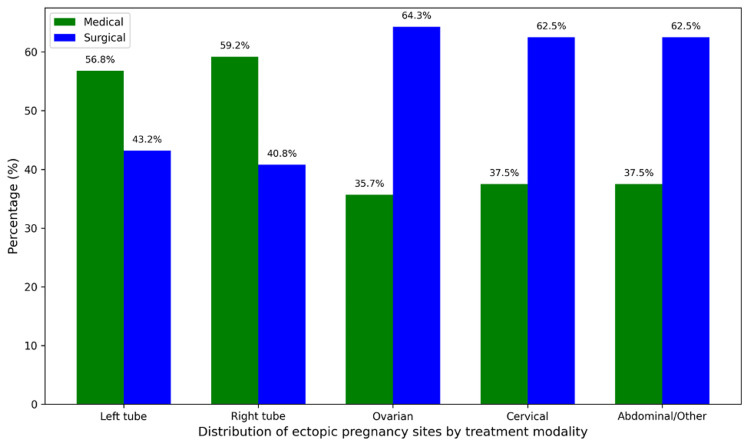
Distribution of ectopic pregnancy sites by treatment modality

In this cohort of 404 women diagnosed with ectopic pregnancy, tubal sites were the most frequently involved, with the left tube affected in 176 cases (43.6%) and the right tube in 152 cases (37.6%). This distribution underscores the predominance of tubal involvement in ectopic pregnancy and highlights the clinical tendency toward surgical management in rarer, non-tubal presentations. Statistical analysis confirmed a significant association between the site of ectopic pregnancy and the type of treatment received (p = 0.0239) (Figure 1).

**Figure 2:**
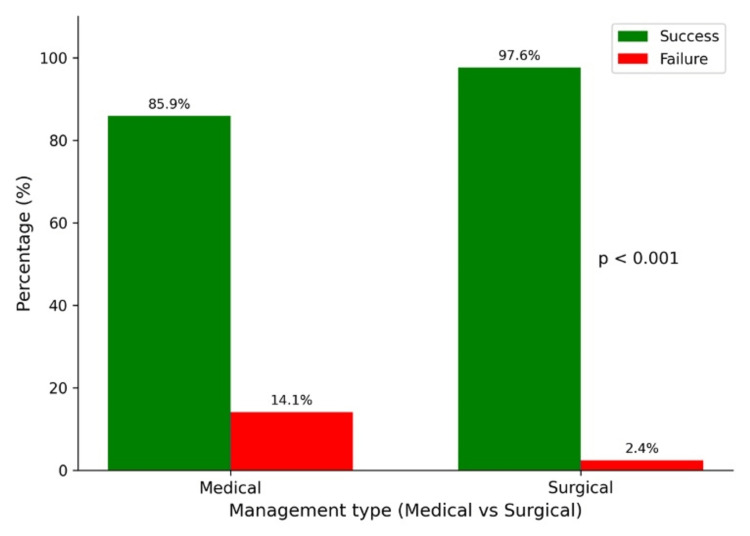
Treatment outcome in patients managed medically vs. surgically

Figure 2 illustrates the comparative treatment outcomes between medical and surgical management of ectopic pregnancy. The success rate was markedly higher in the surgical group, with 97.6% of patients achieving resolution compared to 85.9% in the medical group. Conversely, treatment failure was more frequent among medically managed patients (14.1%) than those undergoing surgery (2.4%). Statistical analysis using the chi-square test demonstrated a significant association between management type and treatment outcome (χ² = 14.5, p < 0.001).

## Discussion

The current investigation reveals that surgical intervention for ectopic pregnancy yields a significantly elevated treatment success rate (97.6%) in comparison to medical management utilizing MTX (85.9%), with a robust statistical correlation (p < 0.001). Individuals who underwent surgical procedures demonstrated an almost fivefold increase in the likelihood of achieving successful treatment outcomes (AOR = 4.85, 95% CI: 2.01-11.7) relative to those receiving medical treatment, demonstrating the superior effectiveness of surgical methods, particularly in instances of advanced or ruptured ectopic pregnancies. These results align with previously published literature indicating that surgical approaches, especially laparoscopic techniques, provide more definitive resolutions and reduce the probability of treatment failure [13,14]. Nevertheless, the advantages associated with higher success rates must be considered in conjunction with the disadvantages of surgical intervention, as patients in this category experienced a substantially prolonged hospital stay (mean 4.8 ± 2.1 days) when contrasted with those undergoing medical therapy (2.4 ± 1.2 days, p < 0.001), reflecting the invasiveness of surgical procedures and the requisite postoperative recuperation. Despite this, the prevalence of post-treatment complications was similar across both cohorts (7.7% vs. 7.3%, p = 0.88), suggesting comparable safety profiles and indicating that advancements in laparoscopic methodologies may have alleviated risks traditionally linked to surgical interventions. Laparoscopic techniques, which constituted nearly 80% of all surgical cases, likely played a pivotal role in achieving these favorable results due to diminished tissue trauma and expedited recovery times compared to open surgical procedures [15]. However, a significant drawback of medical therapy was the increased requirement for further intervention (5.1% vs. 0.8%, p = 0.016; AOR = 6.78, 95% CI: 1.35-33.9), signifying that MTX treatment may not succeed in a subset of patients, thereby necessitating surgical conversion [16].

Furthermore, approximately one-third (28.8%) of patients under medical management necessitated a multidose MTX regimen, highlighting the variability in treatment responses and the importance of vigilant biochemical monitoring. Collectively, these findings indicate that although medical therapy constitutes a significant non-invasive alternative for hemodynamically stable individuals with early, unruptured ectopic pregnancies, surgical intervention, particularly through laparoscopic methods, provides the greatest probability of achieving definitive success while minimizing the risk of complications. Consequently, treatment strategies should be personalized, considering the patient's clinical status, β-hCG levels, and reproductive aspirations, with a multidisciplinary framework ensuring both safety and effectiveness in the management of ectopic pregnancies [17].

The results of this study confirm that tubal implantation is still the most common site for ectopic pregnancies. This aligns with earlier research indicating that over 90% of these pregnancies happen in the fallopian tubes, often due to reasons like previous pelvic infections, tubal surgeries, or issues with tubal motility [18]. Interestingly, both the left and right tubes are nearly equally affected, showing no preference for one side over the other. There’s a notable link between where the implantation occurs and the treatment method used (p = 0.0239), highlighting how crucial the anatomical location is in making management decisions. Non-tubal ectopic pregnancies, like those in the ovaries or cervix, tend to be less responsive to medical treatments, which means they often require surgical solutions [19]. The treatment outcomes further illustrate that surgical management is more effective, boasting a success rate of 97.6% compared to 85.9% for MTX therapy. This higher success rate is consistent with previous studies that emphasize how surgical options, especially laparoscopy, provide immediate and definitive solutions for ectopic pregnancies [20,21]. On the other hand, the lower effectiveness of medical management could be due to factors like elevated baseline β-hCG levels, larger ectopic sizes, or incomplete trophoblastic regression, all of which can hinder the response to MTX. Additionally, the necessity for strict follow-up and varying levels of patient compliance can further impact the success of medical therapy [22]. Despite these challenges, advancements in minimally invasive laparoscopic techniques have made surgery safer and more effective. The strong statistical correlation between the type of treatment and the outcomes (χ² = 14.5, p < 0.001) underscores the reliability of surgical management, particularly in more complex or non-tubal cases.

In our study, baseline characteristics and clinical presentations were comparable between medical and surgical groups, consistent with earlier reports that demographic and obstetric history alone do not determine treatment choice. The key distinction was hemodynamic instability, which was significantly more frequent among surgically managed patients and independently predicted the need for operative intervention. Similar findings have been reported by Keikha F et al. and others, who emphasized that while MTX is highly effective in stable patients, surgery remains the standard in unstable cases or when rupture is suspected [23]. Comparative studies also highlight that laparoscopic surgery offers advantages over laparotomy, including shorter recovery and reduced morbidity, with fertility outcomes broadly comparable to medical therapy [24,25]. Taken together, these results reinforce the principle that patient stability is the decisive factor guiding management, while the choice of surgical technique can further influence recovery and reproductive outcomes.

Strengths and limitations

This study possesses several strengths, including a relatively large sample size of 404 patients, a three-year retrospective design across a tertiary care center, and comprehensive data collection encompassing demographic, clinical, laboratory, imaging, management, and outcome variables, which enhances the generalizability and robustness of the findings. The use of standardized data extraction forms and independent review by two researchers minimized information bias, while multivariate analyses helped identify key predictors of treatment outcomes. However, limitations include the study's retrospective nature, which may be prone to unmeasured confounding and missing data, as well as its single-center design, which could limit external validity. Additionally, long-term fertility outcomes and patient-reported measures were not consistently available, restricting assessment of the broader reproductive impact of medical versus surgical management.

## Conclusions

In this study, both medical and surgical approaches to managing ectopic pregnancy were shown to be safe and effective. Surgical intervention yielded a higher overall success rate, whereas medical therapy provided benefits such as a shorter hospital stay and reduced invasiveness. Choice of management strategy and clinical outcome was influenced by factors including hemodynamic stability, location of implantation, and pelvic pathology history, highlighting the need for individualized planning of care. These results highlight the need for careful patient choice, close follow-up, and prompt management in order to achieve optimal recovery and reduce adverse effects. Future studies would benefit from multicenter, prospective assessment of long-term fertility outcome and further optimization of risk-based management strategies for ectopic pregnancy.

## Appendices

**Table 3:** Data collection form for retrospective study of ectopic pregnancy

Section	Variable	Details/coding/units
Patient demographics	Patient ID	Unique study identifier (anonymized)
	Age (years)	Numeric
	Gravidity	Numeric
	Parity	Numeric
	Previous ectopic pregnancy	Yes/no
	History of pelvic inflammatory disease	Yes/no
	History of pelvic/abdominal surgery	Yes/no; specify type if yes
	Use of assisted reproductive techniques	Yes/no; specify type if yes
Clinical presentation	Abdominal pain	Yes/no; location: unilateral/diffuse
	Vaginal bleeding	Yes/no; mild/moderate/severe
	Syncope / dizziness	Yes/no
	Hemodynamic status at admission	Stable/unstable (SBP < 90 mmHg or pulse > 110 bpm)
	Vital signs	SBP (mmHg), DBP (mmHg), pulse (bpm), temperature (°C)
Laboratory investigations	Hemoglobin (g/dL)	Numeric
	White blood cell count (×10³/μL)	Numeric
	Serum β-hCG level (IU/L)	Day of admission and follow-up values
Imaging findings	Ultrasound performed	Yes/no
	Ectopic site	Tubal (left/right), ovarian, cervical, abdominal, other
	Ectopic mass size (cm)	Numeric
	Presence of free fluid	Yes/no; estimate volume if available
	Signs of rupture	Yes/no
Management details	Management type	Medical/surgical/expectant
	Methotrexate therapy	Single-dose/multi-dose; total dose (mg/m²)
	Surgical approach	Laparoscopic/open
	Type of surgery	Salpingectomy/salpingostomy/other
	Intraoperative findings	Ruptured/unruptured/hemoperitoneum volume (mL)
	Perioperative complications	Yes/no; specify type
Outcome measures	Treatment success	Yes/no (medical: resolution without surgery; surgical: no additional intervention)
	Length of hospital stay (days)	Numeric
	Need for additional intervention	Yes/no; specify type
	Post-treatment complications	Yes/no; specify type
	Fertility outcome (if available)	Subsequent pregnancy yes/no; outcome if known
